# QM/MM Investigation of the Role of a Second Coordination Shell Arginine in [NiFe]-Hydrogenases

**DOI:** 10.3389/fchem.2018.00164

**Published:** 2018-05-15

**Authors:** Andrés M. Escorcia, Matthias Stein

**Affiliations:** Molecular Simulations and Design Group, Max Planck Institute for Dynamics of Complex Technical Systems, Magdeburg, Germany

**Keywords:** hydrogen conversion, enzyme, QM/MM, amino acid substitution, catalysis

## Abstract

[NiFe]-hydrogenases are highly efficient catalysts for the heterolytic splitting of molecular hydrogen (H_2_). The heterobimetallic cysteine-coordinated active site of these enzymes is covered by a highly conserved arginine residue, whose role in the reaction is not fully resolved yet. The structural and catalytic role of this arginine is investigated here using QM/MM calculations with various exchange-correlation functionals. All of them give a very consistent picture of the thermodynamics of H_2_ oxidation. The concept of the presence of a neutral arginine and its direct involvement as a Frustrated Lewis Pair (FLP) in the reaction is critically evaluated. The arginine, however, would exist in its standard protonation state and perform a critical role in positioning and slightly polarizing the substrate H_2_. It is not directly involved in the heterolytic processing of H_2_ but guides its approach and reduces its flexibility during binding. Upon substitution of the positively charged arginine by a charge-conserving lysine residue, the H_2_ binding position remains unaffected. However, critical hydrogen bonding interactions with nearby aspartate residues are lost. In addition, the H_2_ polarization is unfavorable and the reduced side-chain volume may negatively affect the kinetics of the catalytic process.

## Introduction

Hydrogen (H_2_) is among the most important energy carriers in a post-fossil era. The generation of H_2_ as a biofuel from sustainable sources is a versatile alternative to the standard generation process from electrolysis of water which requires elevated temperature and expensive catalyst metals (Holladay et al., 2009; Rodionova et al., 2017). Enzymes from bacteria and microalgae are able to perform the same catalysis at room temperature and standard pressure in the absence of a precious noble metal, and can also catalyze the reverse reaction, the heterolytic cleavage of H_2_. Biological H_2_ conversion has attracted much interest owing to its potential application in a post-carbon based scenario employing H_2_ as an energy storage compound and as a transportable fuel itself (Cammack et al., 2001).

**Figure 1:**
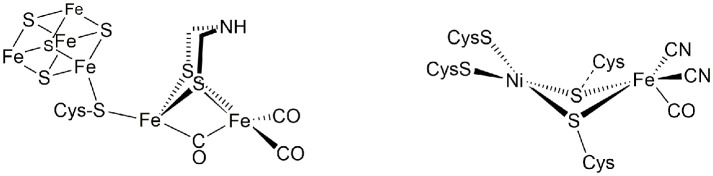
Active sites of [FeFe]- and [NiFe]-hydrogenase enzymes.

These enzymes, the hydrogenases, are classified according to their active site composition as [NiFe]- and [FeFe]-hydrogenases (see Figure 1). The active site of [FeFe]-hydrogenases consists of a μ-carbonyl bridged iron-iron cluster with two additional terminal CO ligands. A bridging azadiothiolate ligand acts as an intermediate proton acceptor during formation of H_2_. The “H-cluster” is connected to a [4Fe4S]-cluster via a bridging cysteine amino acid. The azadithiolate nitrogen in [FeFe]-hydrogenase enzymes acts as an initial site of protonation before the product molecule hydrogen is formed upon reacting with a terminal Fe-bound hydride.

Meanwhile, the active site of [NiFe]-hydrogenases involves two terminal and two bridging cysteine residues and three diatomic inorganic ligands at the iron atom (one carbonyl and two cyanides). The [NiFe]-hydrogenases have a bias toward the heterolytic splitting of H_2_ into protons and electrons. The oxidation of H_2_ makes them useable in a fuel cell (“microbial fuel cell”).

H_2_ can be used in a fuel cell to generate electric power from its oxidation and the reduction of O_2_ to give water. The absence of noble metals and operation conditions at room temperature make [NiFe]-hydrogenase enzymes a system of interest to scientist and engineers, and may act as an inspiration to develop novel bio-inspired catalysts (Du et al., 2007; Cracknell et al., 2008; Santoro et al., 2017). [NiFe]-hydrogenase adsorbed on a pyrolytic graphite electrode catalyzes H_2_ oxidation at a diffusion-controlled rate matching that achieved by platinum (Jones et al., 2002).

**Figure 2:**
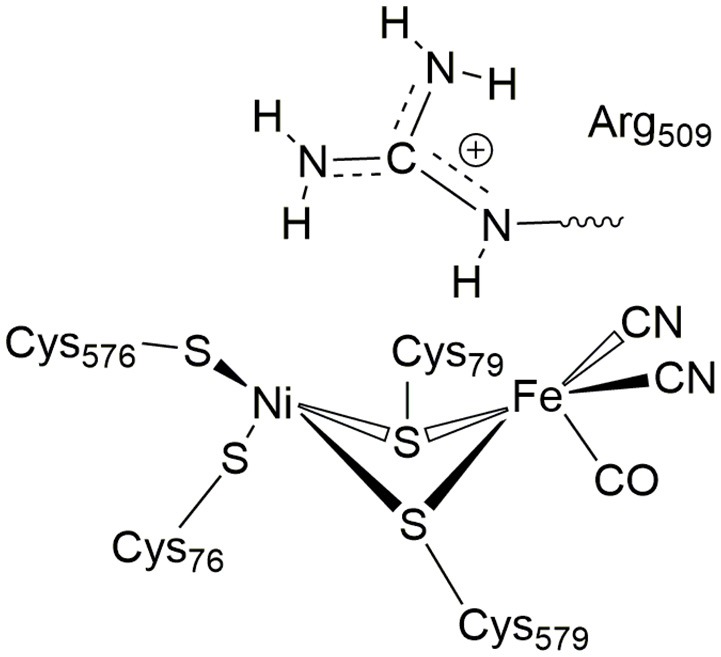
Positioning of a strictly conserved second coordination shell arginine amino acid residue (Arg_509_) above the active site of [NiFe]-hydrogenases (using the amino acid numbering of the [NiFe]-hydrogenase from *E. coli*).

In [NiFe]-hydrogenases, the “as-isolated” oxidized state contains a hydroxide anion (OH^−^) binding between the Ni(III) and Fe(II) ions. During the process of H_2_ activation, nickel shuttles between Ni(III) and Ni(II) oxidation states whereas Fe remains redox-inactive in a 2+ state of oxidation. The catalytic reaction intermediate, Ni-C, is a Ni(III) Fe(II) species with a μ-bridging hydride, but the exact site that acts as the proton acceptor has not been resolved yet. QM and QM/MM calculations have favored one of the terminal cysteines to be the site of protonation (Niu et al., 1999; Lill and Siegbahn, 2009; Hu et al., 2013; Dong and Ryde, 2016; Dong et al., 2018). The ultra-resolution X-ray structure of the fully reduced state of the enzyme, Ni-R, indeed enables to reveal a hydride in the bridging position and one of the terminal cysteines protonated (Ogata et al., 2015b). Recently, however, a non-coordinating amino acid residue was identified to play a major role in H_2_ activation by *E. coli* Hyd-1 (Evans et al., 2016). Substitution of a strictly conserved arginine residue (R509) ~4.4 Å above the active site nickel (see Figure 2) by a charge-conserving lysine led to a >100-fold lower activity in comparison to the wildtype enzyme. This led to the hypothesis of the arginine guanidine group acting as the general base in H_2_ activation (Carr et al., 2016). This would require R509 to at least be fractionally deprotonated and neutral, in order to be able to play a functional role similar to that of a frustrated Lewis pair (FLP) (Stephan and Erker, 2010).

At neutral pH, only lysine, arginine and sometimes histidine possess sidechains with a positive charge. The pKa-value describes the pH-value at which deprotonated and protonated forms are in equilibrium and for arginine, a pKa-value of 12 is usually given in textbooks (Hunter and Borsook, 1924; Berg et al., 2002). At pH < 12, the guanidine nitrogen atom becomes protonated and a positive charge is delocalized via the nitrogen atoms Nη1, Nη2, and Nε (Figure 2). The protein environment can lead to local deviations of the pKa-values of the amino acid side chains due to strong electrostatic interactions with other fully or partially charged groups as well as the polarity or dielectric constants of the medium that surrounds them. pKa-values of catalytic amino acids in or near the active sites of enzymes may be significantly perturbed by more than 2 units due to structural details and the energetics of the reactions that they catalyze (Harris and Turner, 2002). For arginine, however, no detectable shifts in pKa-values were ever reported and, for example, all buried 25 arginine residues in the staphylococcus nuclease remained in the charged state (Harms et al., 2011). Significant perturbations of pKa-values of arginine residues were only found from free energy perturbation calculations from MD trajectories of an arginine residue in a highly hydrophobic membrane environment (Yoo and Cui, 2008). Only when positioned close to the center of the bulk lipid membrane, an effective pKa-value of 7.7 could be obtained. Thus, significant populations of both the protonated and the neutral forms are only possible near the center of the strongly hydrophobic environment. The protein environment surrounding the conserved arginine in [NiFe]-hydrogenases is however far from being hydrophobic and strong (negative) electrostatics from aspartate residues dominate instead.

In this work, we investigate the possible involvement of both neutral and positively charged R509 in the heterolytic splitting of H_2_ by *E. coli* Hyd-1, using QM/MM calculations with various sizes of QM regions. The charge distribution and the energetics of protonation of R509 is almost identical to that of a free arginine residue. Energetically, a simultaneous protonation of R509 and a proton transfer to a nearby aspartate residue is not favorable and the terminal cysteine residue C576 is the preferred proton acceptor. In the R509K mutant enzyme, structural parameters and the charge distribution are only affected to a minor degree. When substrate H_2_ binds to the wildtype enzyme, it is slightly polarized by R509, which facilitates the heterolytic splitting and the proton transfer to the nearby terminal cysteine. This effect is absent in the mutant enzyme. Moreover, R509 is expected to exhibit very low flexibility, as it forms strong hydrogen bonds with surrounding aspartate residues. Thus, we can suggest a dual role of the arginine in the second coordination shell of the hydrogenase enzyme: (i) an electronic function by polarizing the substrate H_2_, and (ii) a structural role by strong electrostatic interactions with negatively charged aspartate amino acid residues and displaying reduced conformational flexibility. By contrast, K509 is not able to form such interactions and it is thus expected to display a higher flexibility than R509, which may kinetically hinder hydrogen conversion in the mutant enzyme.

## Computational details

The initial coordinates of the wildtype and R509K variant of *E. coli* Hyd-1 (hereafter referred to as EH1 and K-EH1) were taken from the crystal structures 5A4M and 4UE3, respectively (Evans et al., 2016). In these structures, the active site is in the oxidized “ready” Ni-B state. The oxygen atom coordinated between the Ni and Fe ions (in both protein structures) as well as oxidized C576 (in 5A4M) were thus removed to meet the functional form of the active site in the Ni-SIa state (see Figure 2). The PDB2PQR suite of programs was used to check the orientation of the side chains of Asn, Gln, and His. We used the PROPKA module of PDB2PQR to assign the protonation states of titratable residues (Dolinsky et al., 2004, 2007; Li et al., 2005; Bas et al., 2008). Both subunits (L: large subunit, and S: small subunit) of the enzymes were taken into account during the PDB2PQR procedure. After protonation, the heteroatoms Ni and Fe (including its CO and CN^−^ ligands) ions of the active site of the L subunit as well as the [FeS]-clusters of the S subunit were inserted at their respective crystal-structure positions. Then, the protonation states and side chain orientations were carefully checked by visual inspection, and potential shifts of the PROPKA-calculated pKa-values due to the presence of the [NiFe] and [FeS] structural motifs were qualitatively assessed for a final assignment of the protonation states of the surrounding residues. Thereafter, the S subunit was removed, while the L subunit was retained for subsequent QM/MM calculations. Moreover, all crystal waters of the latter were deleted except for the active-site water molecules (EH1_L_: 9; K-EH1_L_: 10) (Evans et al., 2016). The protonation states of all EH1_L_ and K-EH1_L_ residues were identical. All acidic residues were negatively charged except for D67, D350, D574, and E73, which were protonated. All lysine and arginine residues were positively charged. Histidine residues were either singly protonated at Nε (H30, 83, 117, 119, 122, 189, 220, 229, 351, 364, 457, and 514), singly protonated at Nδ (H421, 571, and 582), or doubly protonated (H205). All cysteine residues coordinating to metals were deprotonated (C76, C79, C576, and C579).

EH1_L_ and K-EH1_L_ were used as starting structures in subsequent QM/MM calculations with different QM regions as described below.

### QM/MM investigation of the R509 protonation state and its involvement in proton transfer

**Figure 3:**
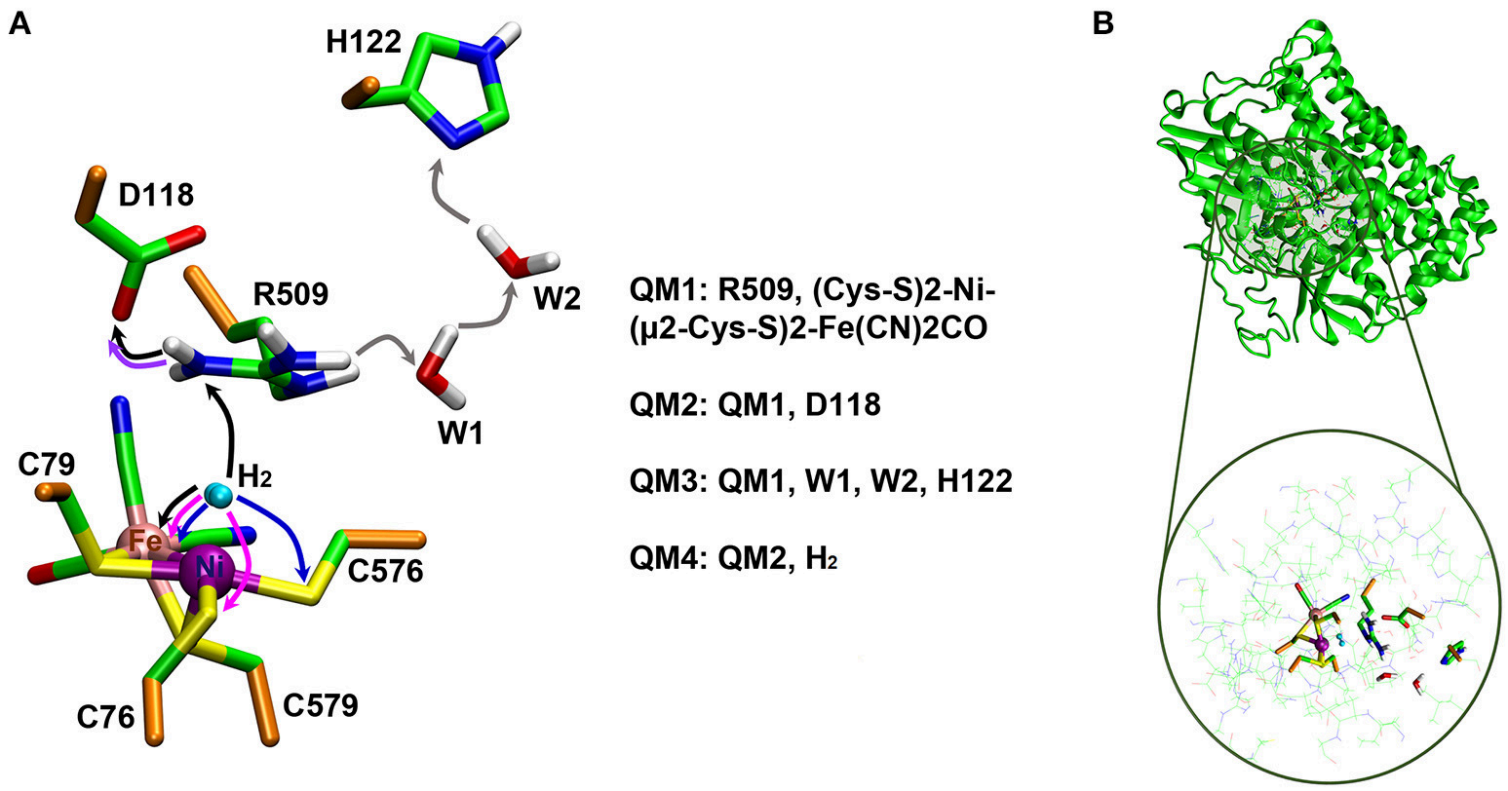
**(A)** QM regions (QM1-QM4) used in the QM/MM calculations. QM/MM boundary (Cα-Cβ) atoms are shown in orange. Reaction mechanisms investigated in this study are indicated in arrows; a unique color is used for each mechanism (e.g., black arrows are used to highlight an R509^+^-assisted H_2_ activation). **(B)** Representative structure of the simulation system. The active region in the QM/MM calculations is enlarged at the bottom with the atoms of the QM1-QM4 regions highlighted. See the text for more details.

Two QM/MM optimizations of EH1_L_ were carried out with R509 being either in neutral (R509^0^) or protonated (R509^+^) form. Among the nitrogen atoms of the guanidinium group of R509, the Nη1 atom is proposed to be the one potentially involved in H_2_ activation since it is closer to the NiFe active site (Evans et al., 2016). This nitrogen atom was therefore chosen as deprotonation target to give R509^0^. The QM region (hereafter referred to as QM1 region) consisted of the side chain of R509^+/0^ as well as the Ni and Fe ions with their first coordination sphere ligands (CO, two CN^−^ groups, and the side chains (thiolate groups) of the four metal-coordinating cysteine residues) (see Figure 3A). The rest of the system (including the active-site water molecules) was treated at the MM level. The total charge of the QM1 region was −1 for R509^+^ and −2 for R509^0^. All atoms within 6 Å of the QM1 region were unconstrained during QM/MM optimization whereas the positions of the more distant atoms were kept fixed (see Figure 3B). The QM/MM calculations were performed with the ChemShell^1^ package (Sherwood et al., 2003; Metz et al., 2014) (version 3.7). The TURBOMOLE (Ahlrichs et al., 2011) (version 6.6) and DL POLY (Smith and Forester, 1996) (version 4.08) packages were used as QM and MM interfaces, respectively. The DL-FIND optimiser module of ChemShell was used for the optimizations (Kästner et al., 2009). The electrostatic interaction between the QM1 region and the surrounding partial charges was treated using the electrostatic embedding scheme with charge shift correction (Bakowies and Thiel, 1996; de Vries et al., 1999). Hydrogen link atoms were used to saturate the valencies at the covalent bonds crossing the QM/MM boundary (see Figure 3A; Sherwood et al., 1997). DFT was used to describe the QM1 region while the MM region was described by the CHARMM27 force field (MacKerell et al., 1998; Mackerell et al., 2004). Geometries were optimized using BP86 (Slater, 1951; Vosko et al., 1980; Perdew, 1986a,b; Becke, 1988) as the DFT functional with the def2-TZVP (Weigend and Ahlrichs, 2005) basis set. The calculations were sped up by using the resolution-of-identity (RI) approximation (Eichkorn et al., 1995, 1997). Single-point energy calculations at the BP86-D3 level were performed to check the influence of dispersion (Slater, 1951; Vosko et al., 1980; Perdew, 1986a,b; Becke, 1988; Grimme, 2006). For further validation of consistency, single-point calculations were also carried out using the density functionals TPSSH (Tao et al., 2003) and B3LYP (Slater, 1951; Vosko et al., 1980; Becke, 1988, 1993) with dispersion corrections (Grimme, 2006). From the QM/MM energies of EH1_L_-R509^0^ and EH1_L_-R509^+^, the energy for the deprotonation of R509 was calculated.

QM/MM calculations were also performed to investigate the ability of R509^+^ to transfer a proton to residues in its close surrounding, and to get insight into potential deprotonation mechanisms leading to formation of R509^0^. Two different mechanisms were considered: (i) a proton transfer from the R509^+^:Nη1 atom to D118:Oδ, and (ii) a water-mediated proton transfer from R509^+^:Nη2 to H122:Nδ. The reactant (R509^+^) and expected product for such reaction mechanisms were optimized to calculate the respective reaction energies. The calculations were carried out using the same QM/MM setup described above. The only difference was in the QM region. For the proton transfer to D118, the side chain of the latter was also included as part of the QM region, whereas for the water-mediated proton transfer the side chain of H122 and two active-site water molecules were included in the QM region. The resulting QM regions are referred to as QM2 (with a charge of −2) and QM3 (with a charge of −1), respectively (see Figure 3A). Initially, the reactant state was optimized. Then the optimized reactant structures were used as starting points to manually build the products (by relocation of the respective protons) and optimize them.

### QM/MM investigation of the thermodynamics of H_2_ activation

We also performed QM/MM calculations to compute the thermodynamic profiles of the EH1_L_ catalyzed H_2_ oxidation with both R509^0^ and R509^+^ acting as a base in the reaction. The potential ability of R509^+^ to mediate H_2_ dissociation was considered to be assisted by a strong R509^+^:Nη1-D118:Oδ hydrogen bond, which could make R509^+^:Nη1 slightly nucleophilic and thus enable a double proton transfer (H^+^ → R509^+^ → D118) reaction. For comparison, we computed the reaction energies with C76 and C576 as the H_2_ proton acceptors, for both EH1_L_ and K-EH1_L_. The reactant [(K-)EH1_L_· H_2_] complexes were optimized first. The latter were built by manual docking of H_2_ into the EH1_L_ and K-EH1_L_ active sites; H_2_ was positioned between Ni and either R509 or K509 and then fully optimized without any additional constraints. Again, the optimized reactants served as starting points to build the products, which were then also fully optimized. This time the QM/MM optimizations were carried out using a QM region (referred to as QM4) which includes the QM2 components as well as the H_2_ atoms (see Figure 3A). All geometry optimizations were carried out considering both Ni and Fe to be in a closed-shell low-spin singlet state. Though the spin state of Ni^2+^ is a controversial topic, recent computational and experimental studies on [NiFe]-hydrogenases using advanced and accurate methods (e.g., coupled cluster calculations and subatomic resolution protein crystallography) support the singlet state to be preferred (Bruschi et al., 2014; Delcey et al., 2014; Ogata et al., 2015a,b, 2016; Dong et al., 2017). Moreover, the protonation site of the enzyme during H_2_ splitting as identified by subatomic resolution X-ray crystallography (Ogata et al., 2015b), was perfectly reproduced by computational calculations carried out with Ni in a single state (Dong and Ryde, 2016). For further validation in this regard, we have carried out single point calculations of the optimized geometries with Ni in a triplet state. Unless mentioned otherwise all energies reported in this manuscript are given with respect to the reactant complex and correspond to the computed energies for Ni in a singlet state which is shown to be the ground state (see below).

Atomic charge distributions in the QM region were calculated by the Mulliken population analysis approach in order to allow to make general statements about trend rather than absolute numbers (Mulliken, 1955a,b).

## Results

### Structural parameters and charge distributions

Table 1 compares the structural parameters of the relevant interactions of R509 with the active site as obtained from the QM/MM calculations using the QM regions 1 and 2 (see above) to those obtained from X-ray crystallography. The BP86 functional was shown to give reliable structural parameters in good agreement with experiment for the active site of [NiFe]-hydrogenases (Stein et al., 2001; Stein and Lubitz, 2004) and other transition metal complexes (Bühl and Kabrede, 2006). Overall, the QM(BP86)/MM(CHARMM) calculations reproduce well the interatomic distances corresponding to the non-covalent interactions between R509 and the Ni-Fe catalytic core. This is more difficult to achieve compared to covalent bonding, since structural flexibility and weak packing interactions must be treated equally well.

**Table 1:** Definition of structural parameters of Arg509 coordination in the vicinity of the active site of the *E. coli* [NiFe]-hydrogenase.

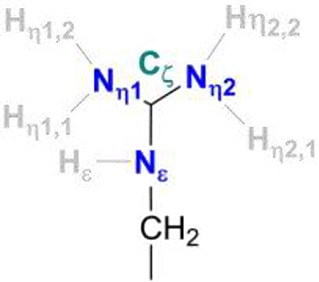	**Distances /Å**	**X-ray**	**QM/MM Arg509^+^ QM1; QM2**	**QM/MM Arg509^0^ QM1; QM2**
	Ni … Nη1	4.44	4.74; 4.48	4.21; 4.48
	Ni … Nη2	5.45	5.71; 5.69	5.37; 5.59
	Ni … Nε	4.89	4.84; 4.93	4.66: 4.76
	Fe… Nη1	4.40	4.66; 4.57	4.28; 4.48
	Fe … Nη2	6.27	6.46; 6.48	6.25; 6.35
	Fe … Nε	5.02	5.10; 5.14	4.97; 4.98
	CN … Nη1	3.22	2.90; 2.93	3.16; 3.11
	CN … Nη2	5.43	5.15; 5.15	5.36; 5.31

*QM/MM optimization results vs. X-ray structural data (PDB code 5A4M)*.

**Figure 4:**
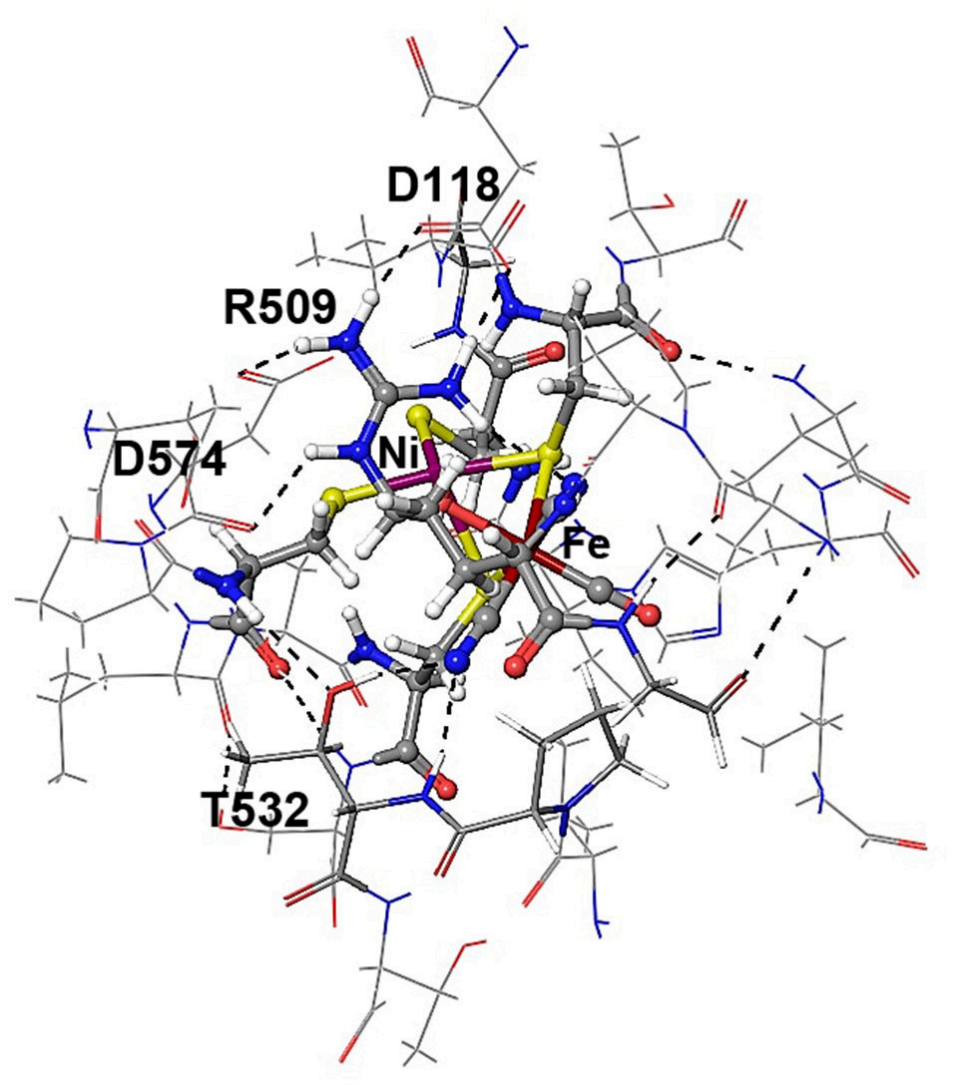
Details of hydrogen bonding interactions of the active site of *E. coli* Hyd-1 with surrounding amino acid residues of the large subunit with a focus on the arginine residue 509. The Nη1 hydrogen atoms form hydrogen bonds with aspartate 118 and one of the cyanide ligands. The Nη2 hydrogen atoms make interactions with the aspartate residues 118 and 574. The terminal cyanide ligands are hydrogen bound to R509 side chain NH_2_ and main chain NH protons; the second cyanide group accepts hydrogen bonds from the OH side chain and the NH main chain protons of Thr532.

The Nη1 amine group forms a hydrogen bond with one of the cyanide ligands of the Fe atom (CN…Nη1 distance ~3 Å) as well as strong electrostatic interactions with the negatively charged aspartate residue D118 (see Figure 4). When D118 is incorporated into the QM region (QM2), the structural parameters are in better agreement with experiment. This shows that an appropriate choice of the QM size is critical for an accurate description of long range interactions in an enzyme.

When R509 is deprotonated, there are only minor structural differences to be seen (Table 1). The variations are within the accuracy of the computational method and give no additional information as to the protonation state of R509 in the crystal structure.

Table 2 provides the partial charges of the R509 atoms as obtained from the QM/MM calculations using the Mulliken population analysis approach (Mulliken, 1955a,b) and the QM regions 1 and 2, and compares them to those calculated for a free arginine. The formation of a delocalized double bond (a partial charge character) makes the central Cζ atom positively charged with 0.45 in a free arginine residue and 0.33 and 0.37 in QM regions 1 and 2, respectively. The Nη1 and Nη2 atoms are overall chemically equivalent in the free arginine with charges of −0.41 and −0.42. In the calculation with the QM1 region, charges of −0.5 and −0.53 indicate a stronger polarization due to interactions with surrounding residues. When D118 is explicitly included in the QM region (QM2), charges of−0.40 and−0.46 are obtained. This shows that the explicit QM electrostatic interaction of residue D118 leads to a slightly chemical non-equivalence of the Nη atoms of R509. The Nε is significantly less negatively charged (−0.22 in free arginine, −0.32 in QM1, and −0.31 in QM2) than the other nitrogen atoms. In the neutral form R509^0^, Cζ becomes significantly less positively charged (+0.25) in free arginine but the effect is less pronounced when embedded in the protein (+0.29 and +0.26 in the different QM regions). Upon deprotonation of Nη1, its charge changes only marginally both in the free residue (by 0.01) and the one in the protein (by 0.03–0.06). For Nη2, on the other hand, the negative charge increases by 0.05 in the free residue and 0.09–0.10 in the protein. Thus, Nη2 becomes more nucleophilic in neutral arginine. In the enzyme, this nitrogen atom is however at a too large distance from Ni (>6 Å) so that a direct involvement in hydrogen activation is not feasible.

**Table 2:** Charge distributions of a free arginine residue and the residue Arg509 of the *E. coli* [NiFe]-hydrogenase in their standard and neutral protonation states.

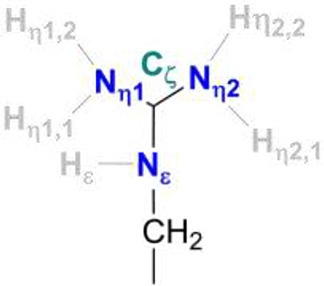	**Atom**	**Free Arg^+^/Arg^0^**	**QM/MM Arg509^+^/Arg509^0^ QM1; QM2**
	Nη1	−0.413/−0.415	−0.498/−0.441; −0.400/−0.433
	Nη2	−0.422/−0.473	−0.527/−0.623; −0.462/−0.560
	Nε	−0.224/−0.285	−0.318/−0.328; −0.313/−0.328
	Cζ	0.447/0.247	0.333/0.289; 0.370/0.258

In conclusion, we cannot report a significant perturbation of the charge distribution in R509 with respect to a free arginine residue. We therefore do not expect this residue to display a significantly perturbed pKa-value.

On the other hand, we calculate the reaction energy for the deprotonation of R509 from the computed QM/MM energies of EH1_L_-R509^+^ and EH1_L_-R509^0^. We assume that the free proton is able to diffuse out of the protein (EH1_L_-R509^+^↔ EH1_L_-R509^0^ + H^+^) by considering the free energy of solvation of H^+^ in water [ΔG_solv_ (H^+^) = −265.9 kcal/mol; (Tawa et al., 1998; Tissandier et al., 1998; Topol et al., 2000; Kelly et al., 2007; Rebollar-Zepeda and Galano, 2012). The results are shown in Table 3, where a positive deprotonation energy indicates the thermodynamic preference for protonated R509^+^. All DFT calculations give consistent results for the thermodynamic equilibrium between R509^+^ and R509^0^. The differences between different functionals are within 4 kcal/mol. The protonated form R509^+^ in the protein is energetically favored by 154–158 kcal/mol. This large deprotonation energy shows that in the [NiFe]-hydrogenase the arginine residue R509 close to the active site is predominantly in its protonated form. Meanwhile, attempts to calculate the energy difference between R509^+^ and R509^0^ with either D118 or H122 as a proton acceptor (see Figure 3) were unsuccessful; reversion to the zwitterionic state of R509 occurred immediately in the QM/MM optimizations involving R509^0^.

**Table 3:** Calculated deprotonation energies of R509 of *E. coli* Hyd-1 using QM/MM calculations in kcal/mol.

**Level of Theory**	**Deprotonation Energy**
BP86	154.1
BP86-D3	156.3
B3LYP-D3	155.5
TPSSH-D3	158

It should be noted that we are not attempting to calculate standard and perturbed pKa-values here, since it requires of a more robust computational approach to be implemented, including e.g., high level electronic structure methods, explicit solvent coordination with a certain number of solvent molecules plus continuum solvation, consideration of entropic contributions, and conformational sampling (Ghosh and Cui, 2008; Rebollar-Zepeda and Galano, 2012; Uddin et al., 2013). It can only be stated that in *E. coli* Hyd-1, the amount of neutral R509 is negligible and the thermodynamic equilibrium is far toward a positively charged residue in its standard protonation state. Thus, the direct involvement of the neutral form of R509 as a strong FLP in H_2_ oxidation appears impossible.

### The substrate bound complex

Ni-SIa is the catalytically active species which performs hydrogen oxidation. In Ni-SIa, H_2_ approaches the Ni site where it is heterolytically cleaved. The hydride occupies the μ-bridging position between the Ni and Fe atoms and one residue in the vicinity must act as a proton acceptor.

Table 4 gives structural data for the H_2_ Ni-SIa complexes corresponding to the EH1_L_-R509^+^ wildtype enzyme and the R509K variant. Attempts to compute structural parameters and reaction energies for EH1_L_-R509^0^ were unsuccessful, since the optimization of the respective reactant complex evolved spontaneously toward the product with a μ-hydride and a protonated arginine. This reinforces our conclusion on the preference of R509 to be in the protonated state.

**Table 4:** Structural data of the H_2_ Ni-SIa complexes of the wildtype *E. coli* Hyd-1 and the R509K variant optimized using the larger QM2 region.

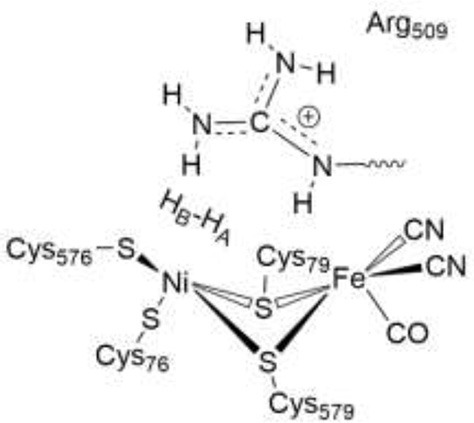	**Distances/Å**	**Wildtype R509^+^**	**Mutant R509K**
	Ni … Nη1	4.4	4.5^a^
	Ni … Nη2	5.5	^b^
	Ni … Nε	4.7	^b^
	Fe… Nη1	4.6	4.9^a^
	Fe … Nη2	6.4	^b^
	Fe … Nε	5.1	^b^
	CN … Nη1	2.9	3.0^a^
**H_2_-BINDING**	Ni… H_A_	1.6	1.6
	Ni … H_B_	1.6	1.6
	Fe … H_A_	2.5	2.4
	Fe … H_B_	3.2	3.1
	Nη1 … H_A_	2.9	3.1^a^
	Nη1… H_B_	2.9	3.0^a^
	C76:S… H_A_	3.6	3.6
	C76:S… H_B_	3.1	3.1
	C576:S… H_A_	2.9	2.9
	C576:S … H_B_	2.7	2.7

a *Distances to the N_ζ_ atom of lysine*.

b *Not applicable*.

In the reactant complex (RC_EH1_), H_2_ is located above and very close to the Ni ion (at 1.6 Å), whereas the distance between the Fe ion and H_2_ is longer (2.5 Å). This is in agreement with previous studies on [NiFe] hydrogenases which show that H_2_ prefers to bind to Ni, rather than to Fe (Ogata et al., 2002; Dong et al., 2017). Upon H_2_ binding, the relevant interatomic distances between the active site and R509 are overall unchanged compared to the Ni-SIa state (see Tables 1, 4). Moreover, structural parameters at the reactant complex are similar for the wildtype and the mutant enzyme. Nickel-nitrogen and iron-nitrogen distances as well as the interactions of H_2_ with nickel, iron, or cysteine residues do not change overall. This indicates that upon the arginine-to-lysine mutation, the active center remains fully assembled and structurally intact to perform the catalytic hydrogen oxidation. This is in agreement with structural and spectroscopic investigations (Evans et al., 2016).

The electronic structure also only changes slightly in the R509K mutant (Table 5). The Nη1 atom becomes less negative (−0.31) and thus less nucleophilic and will be then a weaker proton acceptor when H_2_ is heterolytically splitted. Atomic charges at Ni, Fe and all other active site atoms remain overall unchanged (see Table 5). What becomes apparent by analyzing the partial charges is the fact that in the wildtype enzyme both hydrogen atoms of H_2_ are slightly positively polarized (+0.07). In the lysine mutant, however, the hydrogen atoms become less and oppositely charged, with the hydrogen atom pointing toward the bridging position positive (H_A_, +0.02) and the distal hydrogen between lysine and cysteine negative (H_B_, −0.02). Since H_A_ will become the bridging hydride, H_B_ ought to be accepted by a (negatively charged) proton acceptor. This indicates that the introduction of a lysine residue does not structurally impair the catalytic function, but it reduces the proton affinity of a potential proton acceptor nitrogen atom and at the same time leads to a partial negative charge on the putative protonic species.

**Table 5:** Charge distributions in the H_2_ Ni-SIa complexes of the wildtype *E. coli* Hyd-1and the R509K mutant optimized using the larger QM2 region.

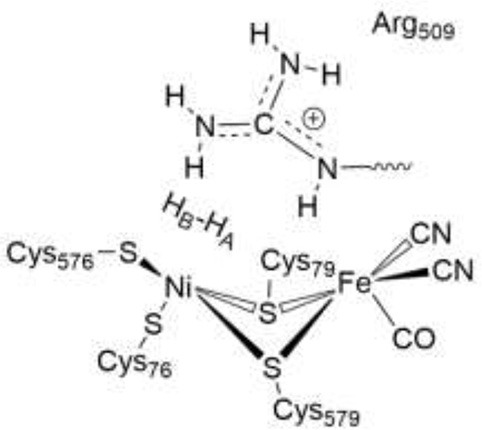	**Atom**	**Wildtype R509^+^**	**Mutant R509K**
	Nη1	−0.402	−0.308^a^
	Nη2	−0.466	^b^
	Nε	−0.296	^b^
	Ni	−0.251	−0.225
	Fe	0.147	0.156
	C76:S	−0.488	−0.475
	C576:S	−0.041	−0.041
	H_A_	0.069	0.020
	H_B_	0.065	−0.018

a *N_ζ_ atom of lysine (K509)*.

b *Not applicable*.

### The thermodynamics of H_2_ oxidation

The QM/MM energies calculated for the binding and heterolytic splitting of H_2_ by EH1_L_-R509^+^ and K-EH1 are shown in Table 6 for a series of different functionals. Since we could not obtain a stationary intermediate for the EH1_L_-R509^0^ H_2_ complex, those energies cannot be reported. All DFT calculations give a very consistent picture of the energetics of H_2_ binding and splitting. This provides a reliable insight into the thermodynamics of H_2_ oxidation and an estimate of the uncertainty of the computed energies.

**Table 6:** Substrate binding energies and thermodynamics of heterolytic H_2_ splitting (in kcal/mol) in the wildtype and R509K mutant [NiFe]-hydrogenase from *E. coli*.

	**BP86**	**BP86-D3**	**B3LYP-D3**	**TPSSH-D3**
**Wildtype**				
H_2_ binding	−2.8	−9.4	−4.4	−8.4
**HETEROLYTIC H**_2_ **SPLITTING. HA-Ni-**μ**H**^−^**-FE**				
**Proton acceptor A**				
Arginine 509^+^	19.3	16.2	13.9	15.1
Cysteine 76	−3.7	−2.2	−9.9	−7.2
Cysteine 576	−8.4	−7.0	−15.3	−12.2
**K509 Mutant**				
H_2_ binding	−1.6	−7.5	−2.7	−6.7
**HETEROLYTIC H**_2_ **SPLITTING. HA-Ni-**μ**H**^−^**-FE**				
**Proton acceptor A**				
Cysteine 76	−11.9	−11.8	−19.1	−16.2
Cysteine 576	−10.1	−9.4	−17.3	−14.4

*The hydride occupies the μ-bridging position between the Ni…Fe atoms*.

As can be seen in Table 6, our calculations indicate that a potential heterolytic splitting of H_2_ involving participation of R509^+^ as a proton acceptor (${\text{H}}_{2}\to R509^{+}\to$D118) is thermodynamically unfavorable by 13.9–19.3 kcal/mol. By contrast, C76 and C576 are found to be able to act as proton acceptors and mediate this process favorably, the reaction being exothermic by 3.7–9.9 and 7.0–15.3 kcal/mol, respectively. At all levels of theory evaluated protonation of C576 is thermodynamically more favorable than that of C76; the reaction energy associated with C576 is 4.7–5.4 kcal/mol lower in comparison to C76. This is in line with previous computational and X-ray crystallography studies on [NiFe]-hydrogenase from *Desulfovibrio vulgaris* Miyazaki F, which show that protonation of C546 (the equivalent for C576 in EH1) is preferred over the other coordinating cysteines (Ogata et al., 2015b; Dong and Ryde, 2016).

**Figure 5:**
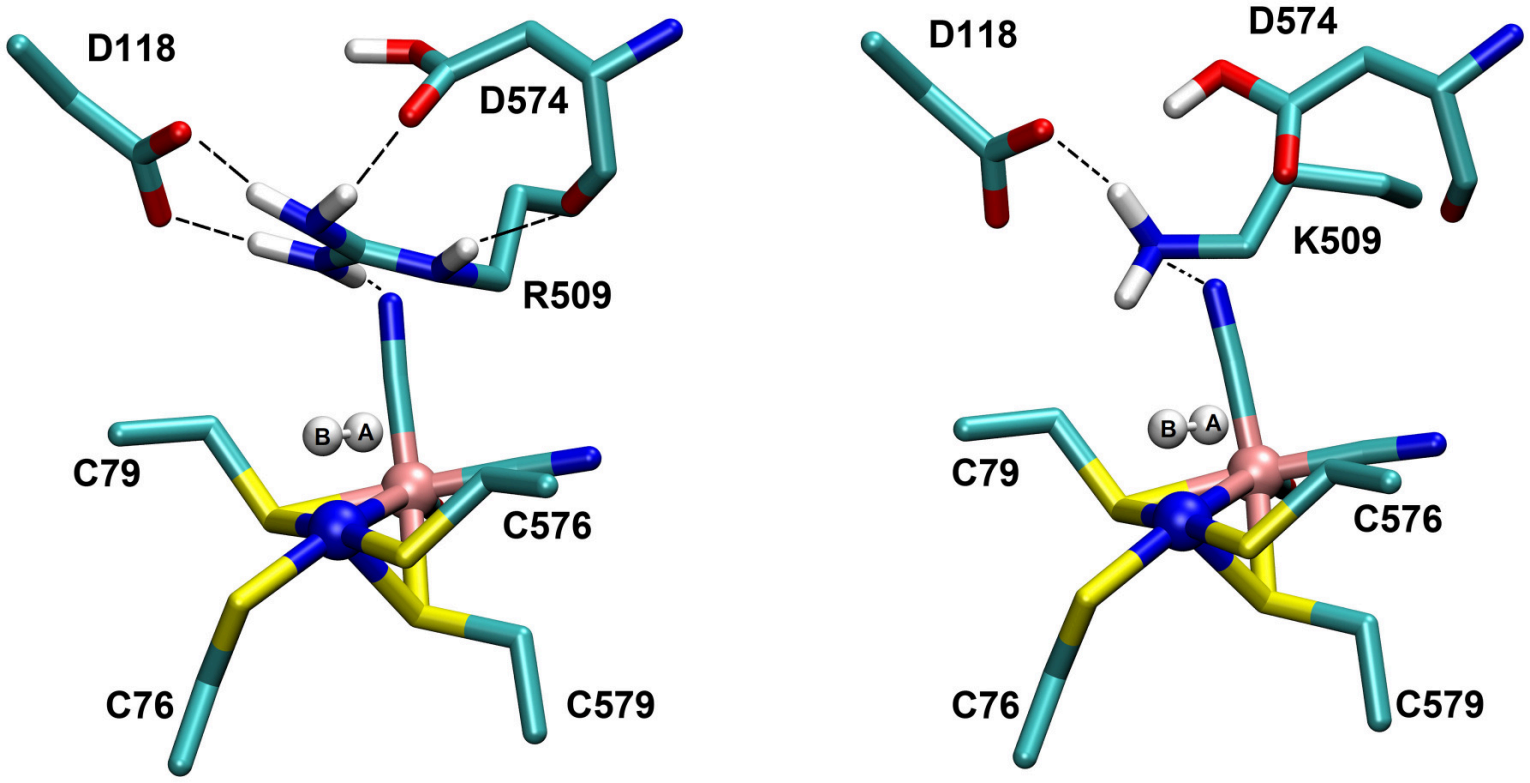
Details of the substrate H_2_ binding in the Ni-SIa state of the catalytic site of the [NiFe]-hydrogenase. **(Left)**: In the wildtype *E. coli* Hyd-1, the arginine residue 509 makes strong hydrogen bonding interactions with the aspartates 118 and 574. **(Right)**: In the R509K mutant, explicit hydrogen bond formation is not given and the electrostatic interactions with the aspartate residues are less pronounced.

The structures of the optimized H_2_…Ni-SI_a_ complexes are shown in Figure 5. Apart from a different positioning of the H_2_:H_B_ atom (see Figure 5), there are only a few structural differences between the optimized product complexes. In PC76_EH1_ (protonated cysteine C76), the orientation of the side chain of the residue E28 (located in the MM region) is different and is better stabilized by the surrounding residues (via hydrogen bonds) in comparison to both PC576_EH1_ (protonated cysteine C576) and PC509_EH1_ (product complex for a proton transfer to R509^+^). This is also true when comparing PC76_EH1_ and RC_EH1_. Therefore, the hydrogen bonding interactions of E28 in PC76_EH1_ are considered to be important for the exothermic formation of this product, which is supported by the lower value of the MM energy component with respect to that for RC_EH1_ (see Supplementary Material for a detailed analysis of the QM and MM energy contributions to the QM/MM energies). (Senn and Thiel, 2009; Escorcia et al., 2017) Meanwhile, PC509_EH1_ differs from PC76_EH1_ and PC576_EH1_ regarding geometry and orientation of the side chain of R509^+^. The characteristic planar geometry of the guanidinium group is distorted in PC509_EH1_. In addition, the hydrogen bond interactions with the surrounding aspartate residues (D118 and D574) are overall weaker. Together these terms may contribute significantly to the endothermic formation of PC509_EH1_, as given by the higher value of the QM energy component in comparison to PC76_EH1_ and PC576_EH1_ (see 'Supplementary Material).

According to these results, R509 is not expected to be directly involved in the reaction mechanism of the H_2_ activation by EH1. Instead, we propose this residue to be important for H_2_ activation by guiding its access to the active site, promoting its binding to nickel and facilitating its polarization. Our conclusions are based on the QM/MM calculations with K-EH1_L_. As can be seen in Table 6, H_2_ activation by K-EH1_L_ is also thermodynamically feasible with either C76 or C576 acting as a base. The computed reaction energies also suggest the process to be thermodynamically comparable with respect to the EH1 wildtype enzyme. This shows that a mere thermodynamic argumentation cannot explain the high activity of EH1 and the >100-fold reduction in K-EH1_L_.

Also, the binding energy of H_2_ is favored by 1.2 kcal/mol in the wildtype EH1. As described above, the charge analysis showed that the H_2_:H_B_ atom is more polarized in RC_EH1_ than in RC_K−EH1_, with an atomic charge value of 0.07 and −0.02, respectively (Table 5). Considering this and the similar negative charge of the sulfur atoms of both C76 and C576, the H_2_ splitting is expected to be kinetically favored (i.e. with a lower energy barrier) in EH1.

The most important structural differences between EH1 and K-EH1 are found to be in the immediate vicinity of the R509 and K509 residues. The former is strongly stabilized by the surrounding aspartate residues through hydrogen bond interactions (Figure 5), which hold R509 in place and may make this residue more rigid in comparison to lysine. The smaller spatial extension and a potential higher degree of flexibility of the side chain of K509 may account for a weaker binding of H_2_ as well as a correct positioning and polarization of the latter in the wildtype enzyme. The investigation of the effect of the flexibility of the side chain on the kinetics of the H_2_ splitting will require extensive QM/MM MD simulations with a sufficient degree of conformational sampling.

All energies discussed up to this point were obtained with Ni in a low-spin singlet state (*S* = 0).

The spin state of the EPR-silent Ni^2+^ intermediate state is still a controversial issue. Recent computational studies on [NiFe]-hydrogenases using state-of-the-art methods (e.g., coupled cluster calculations and DMRG) support the singlet state to be preferred over the triplet in a cluster model of the active site (Dong et al., 2017). BP86 gave a low spin Ni(II) state for NiSI (Stein et al., 2001; Stein and Lubitz, 2004) and was shown to be close to the DMRG results in terms of spin state splitting energies. Meanwhile, B3LYP gave reasonable thermodynamics for H_2_ splitting but did not perform well for triplet *vs*. singlet Ni(II) spin state energies (Dong et al., 2017). With the B3LYP functional, a high spin Ni(II) was found to be the ground state in an earlier study (Pardo et al., 2006). For Ni(II) tetrathiolate complexes, the singlet-triplet energy splitting is very sensitive to the amount of exact Hartree-Fock exchange. A reduction to 0.15 in B3LYP^*^ gave an improved description of the relative spin state ordering for Ni(II)S4 model complexes and [NiFe]-hydrogenase active site models (Bruschi et al., 2004). On the other hand, the TPSSH functional with 0.1 of exact exchange gave reliable structural parameters and bond energies for a set of 80 transition-metal-containing complexes. Furthermore, TPSSH provided reliable energies when tested against typical bioinorganic reactions including spin inversion and electron affinity in iron–sulfur clusters, and breaking or formation of bonds in iron proteins and cobalamins (Jensen, 2008). Thus, we have additionally carried out QM/MM calculations for the thermodynamics of H_2_ splitting with Ni(II) in a triplet state (*S* = 1).

We compare the relative spin ordering of singlet and triplet spin states for the BP86, B3LYP, and TPSSH functionals. As shown in Table 7, the results from all functionals are absolutely consistent and indicate that the reactivity on the singlet state spin surface is favored over the triplet state surface for both the wildtype and the mutant enzyme, by 13–23 kcal/mol.

**Table 7:** Singlet-triplet spin state splitting energies (in kcal/mol) from QM/MM calculations.

	**BP86**	**BP86-D3**	**B3LYP-D3**	**TPSSH-D3**
**Wildtype**				
RC	22.7	22.7	20.9	21.6
PC76	19.2	19.1	15.4	15.5
PC576	18.7	18.7	13.2	13.7
**K509 Mutant**				
RC	22.8	22.8	21.1	21.8
PC76	20.6	20.5	16.6	16.8
PC576	20.3	20.3	15.0	15.6

*A positive energy indicates the singlet state of Ni(II) to be the ground state*.

*RC: reactant complex (H_2_@Ni-SIa complex). PC76: protonated cysteine C76. PC576: protonated cysteine C576*.

## Conclusions

Hydrogen oxidation by *E. coli* Hyd-1 was investigated by QM/MM calculations. Substitution of a highly conserved arginine amino acid residue by a charge conserving lysine does not affect the structural parameters and the electronic structure of the active site. The active site is fully assembled and pre-formed for catalysis. The introduction of lysine, however, leads to an unfavorable polarization of the substrate H_2_ and makes proton transfer to a negatively charged terminal cysteine kinetically impaired. This explains the reduction of activity by two orders of magnitude in the K-EH1 mutant enzyme. It was initially suggested that a neutral arginine R509^0^ might directly be involved in catalysis and act as a FLP for proton acceptance.

FLPs were identified to be highly effective in activating a variety of small molecules and prompted strong interest in their investigation, e.g., the activation of molecular hydrogen in the absence of a transition metal catalyst by a FLP was also reported (Stephan and Erker, 2010). The concept of FLPs has also been applied to the design of model systems for the active sites of the transition metal-containing hydrogenases. DuBois, Bullock, and co-workers (Raugei et al., 2012) developed enzyme model systems that combine a metal center with non-coordinating amine donor ligands. These pendant, neutral amine groups act in concert with the Ni center to give rise to electrochemical H_2_ oxidation and the authors directly note the analogy to FLPs.

In the [NiFe]-hydrogenase enzyme, we cannot verify the existence of a neutral arginine amino acid residue close to the active site. This would be possible only if the pKa-value of that residue was strongly perturbed by the interactions with the protein environment. According to our findings, the charge distribution of R509 in the enzyme is very close to that of a free arginine amino acid residue and the deprotonation energy too high to enable generation of a neutral arginine. In biological catalysis, it is the positively charged side-chain guanidinium group that is often utilized as an electrophilic catalyst with a very high pKa-value (≥12). In the heterolytic cleavage of H_2_, a hydride occupies the μ-bridging position between the Ni and Fe atoms. Rather, a nucleophilic proton acceptor is necessary to take up the product proton. The positively charged R509 residue can still facilitate H_2_ splitting via polarization of the latter due to interactions with the partially negatively charged (nucleophilic) Nη1 atom.

**Figure 6:**
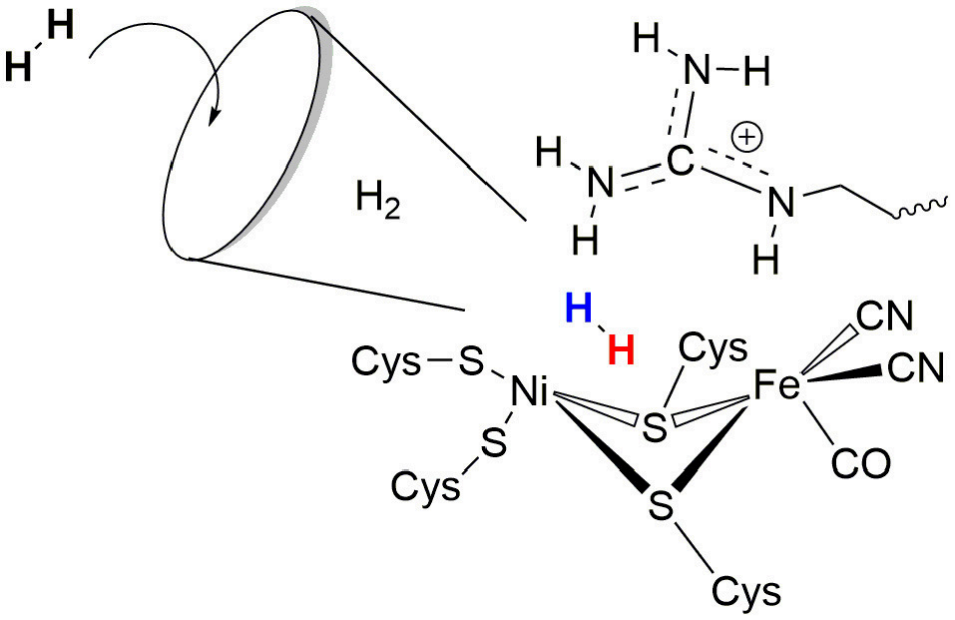
Schematic representation of the role of arginine 509 in hydrogen binding and oxidation by [NiFe]-hydrogenases.

The role of the conserved arginine in hydrogenases is thus three-fold: (i) strong electrostatic interactions with nearby aspartate amino acid residues enable an easy H_2_ access to the Ni atom with an access channel radius of ~4Å (see Figure 6); (ii) the arginine assists the positioning and polarization of H_2_ to enable a swift proton transfer to one of the terminal cysteines; and (iii) the strong electrostatic interactions with the protein environment keep the arginine in a rigid position and obstruct any conformational changes which otherwise might impede catalysis (see Figure 5).

## Author contributions

MS: designed and initiated the project; AE: performed the calculations; MS and AE: analyzed and interpreted the data, wrote the manuscript, and approved the final version.

### Conflict of interest statement

The authors declare that the research was conducted in the absence of any commercial or financial relationships that could be construed as a potential conflict of interest.

## Footnotes

^1^ChemShell, a Computational Chemistry Shell, see www.chemshell.org
