# Supplementary material for: QM/MM Investigation of the Role of a Second Coordination Shell Arginine in [NiFe]-Hydrogenases
Source: Front Chem. 2018 May 15;6:164. doi: 10.3389/fchem.2018.00164 (PMC5962704; doi:10.3389/fchem.2018.00164)
Supplement: Supplementary file 1 [file Data_Sheet_1.PDF]

QM/MM Investigation of Second Coordination Shell Residues in [NiFe]-Hydrogenases

Andres M. Escorcia, Matthias Stein

Supporting Information

Detailed analysis of MM and QM contributions to the QM/MM energies.

Note: MM energy is the same in all single point calculations.

Table RS1. QM and MM energies of H_2 heterolytic splitting by EH1.

Proton acceptor	BP86		BP86-D3	B3LYP-D3	TPSSH-D3
	QM	MM	QM	QM	QM
Arginine 509^+	17.2	2.1	14.1	11.8	13.0
Cysteine 76	3.1	-6.8	4.6	-3.1	-0.4
Cysteine 576	-10.9	2.5	-9.5	-17.8	-14.7

Table RS2. QM and MM energies of H_2 heterolytic splitting by K-EH1.

Proton acceptor	BP86		BP86-D3	B3LYP-D3	TPSSH-D3
	QM	MM	QM	QM	QM
Cysteine 76	10.5	-22.4	10.6	3.3	6.2
Cysteine 576	-13.3	3.2	-12.6	-20.5	-17.6
